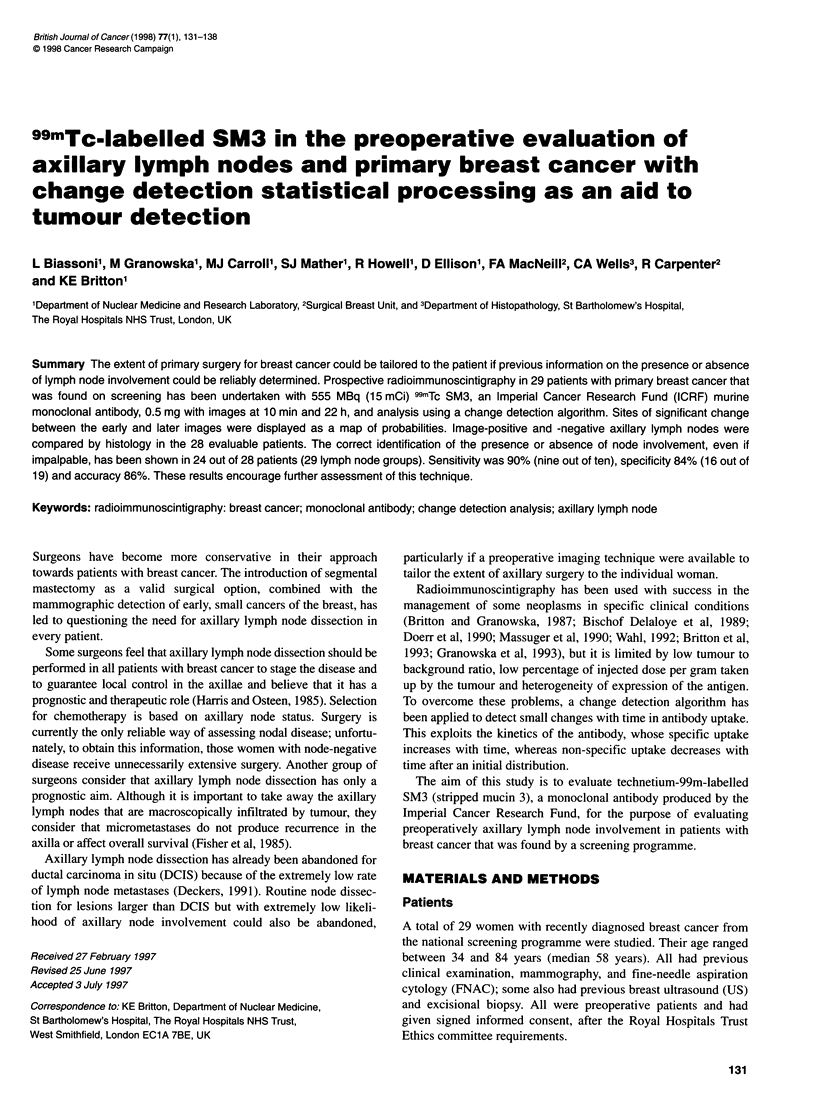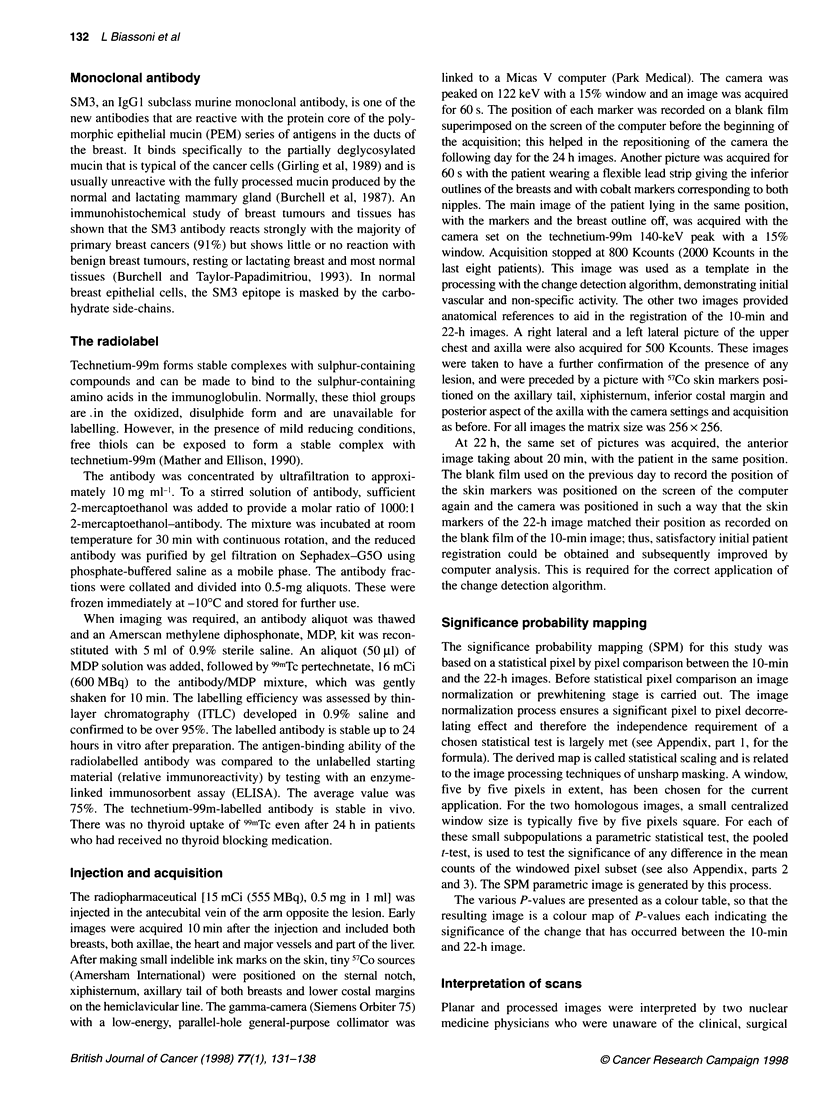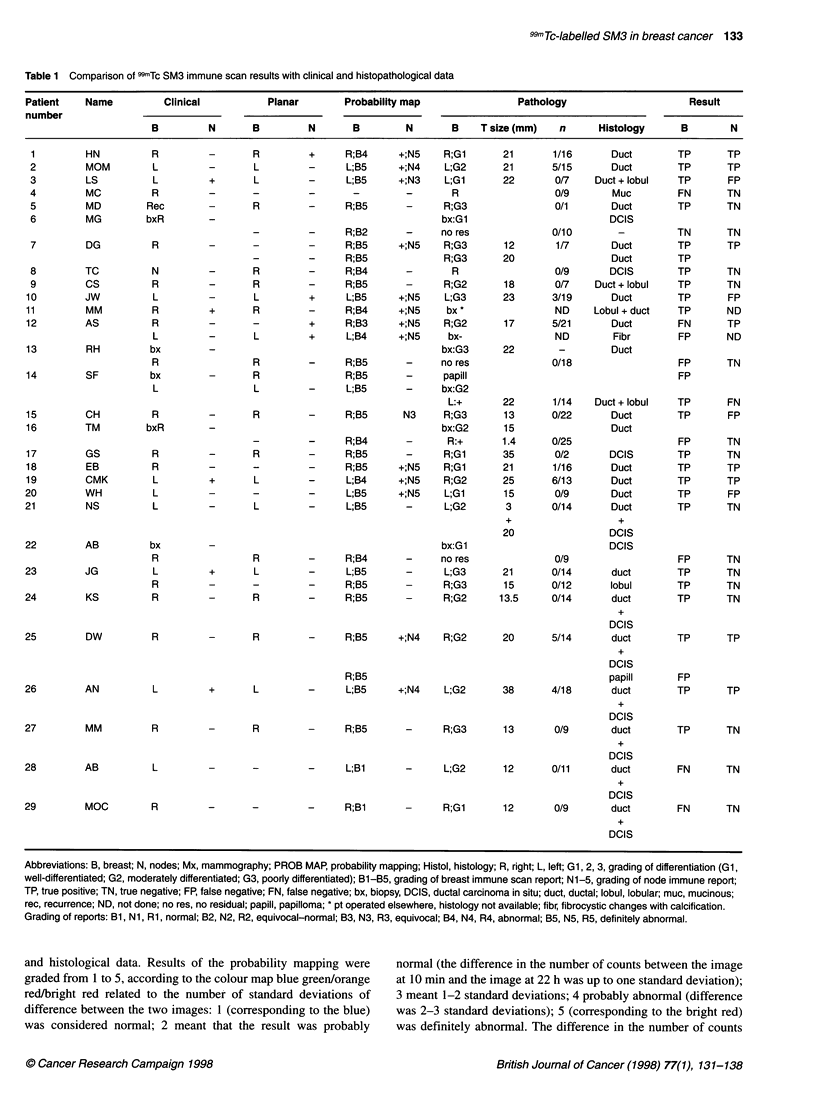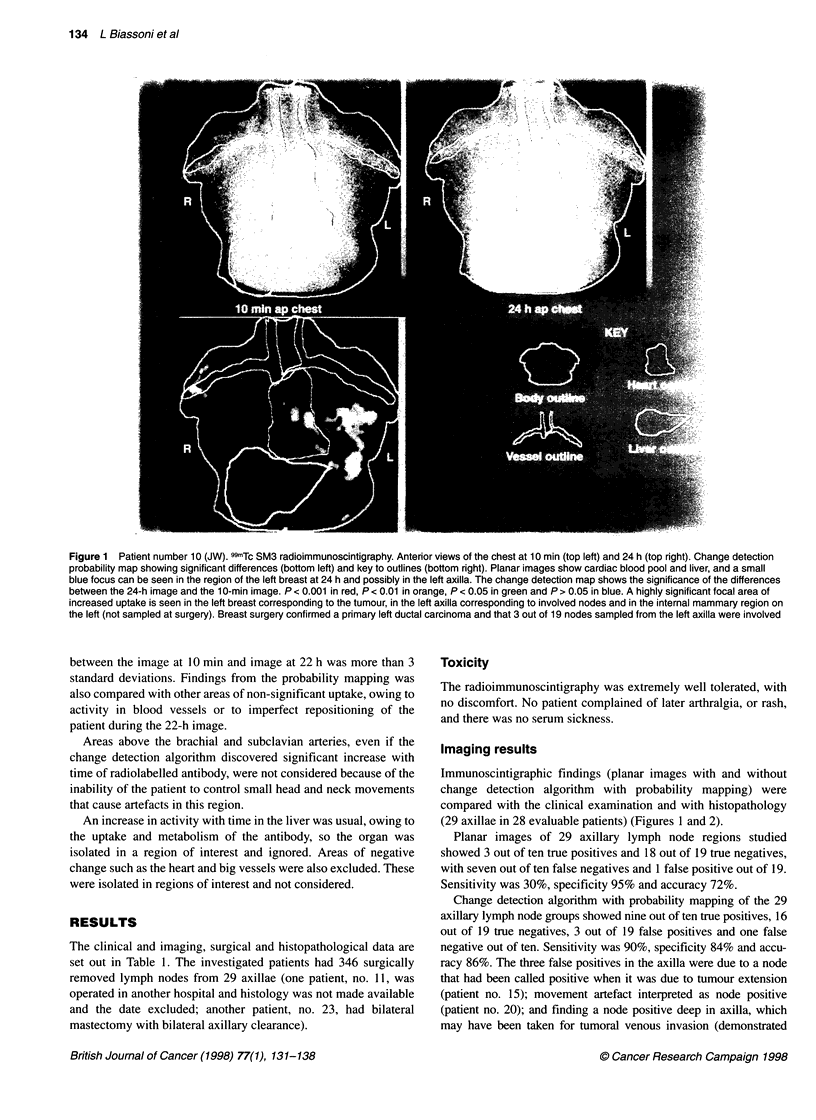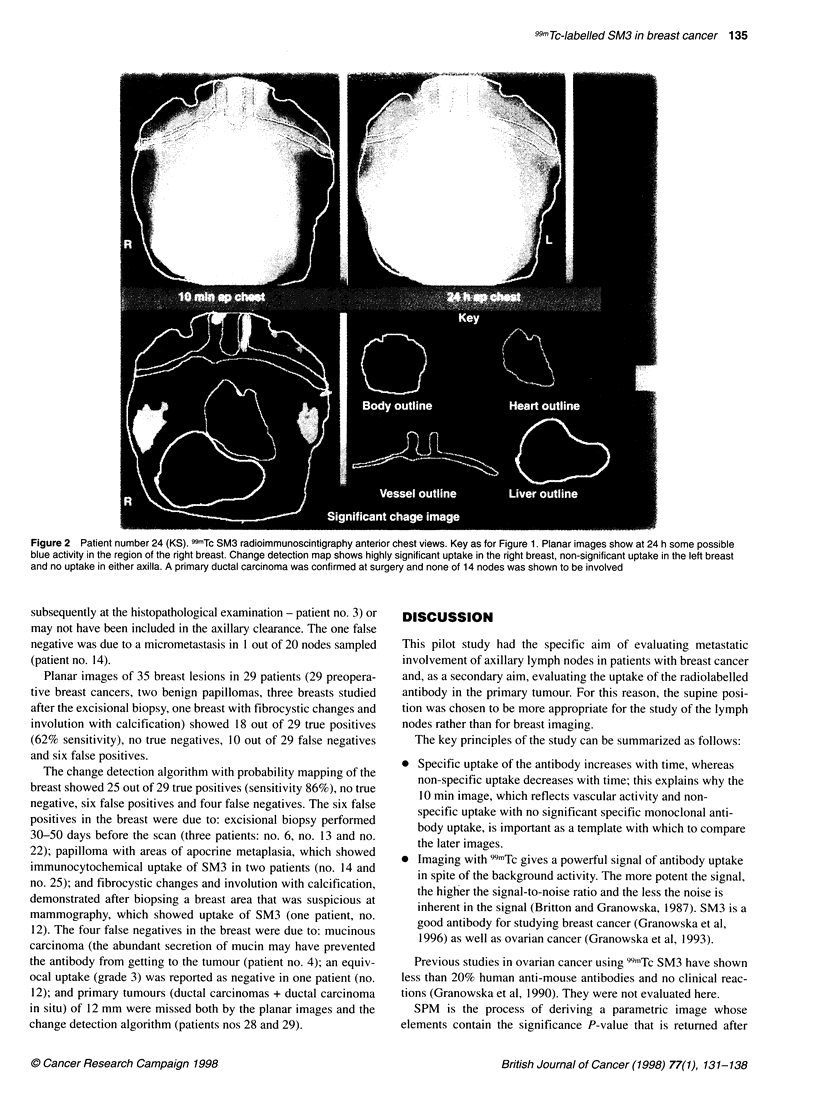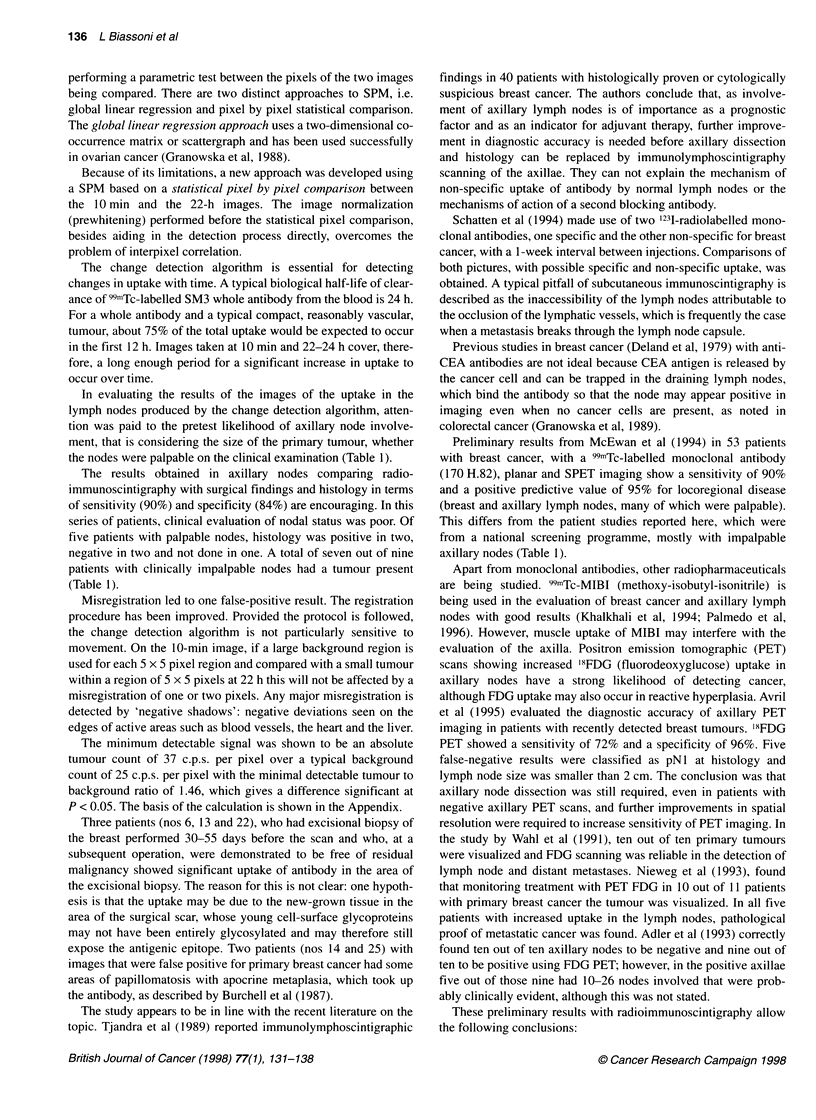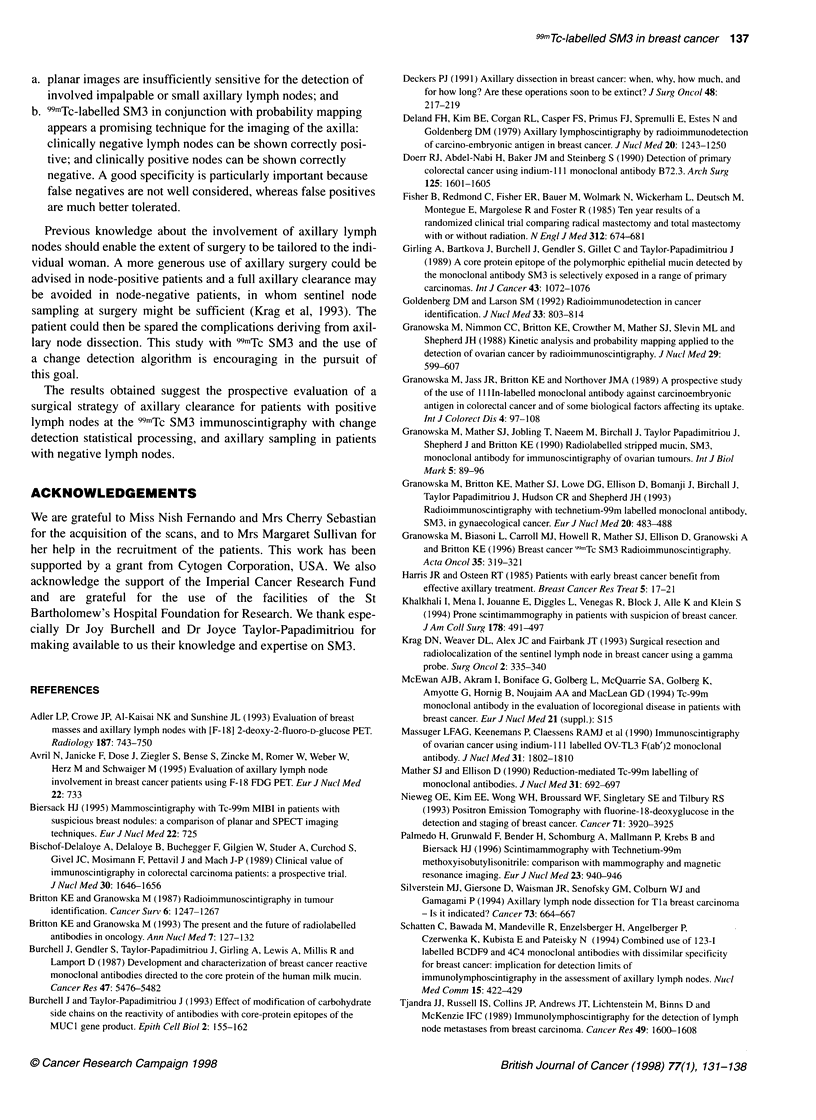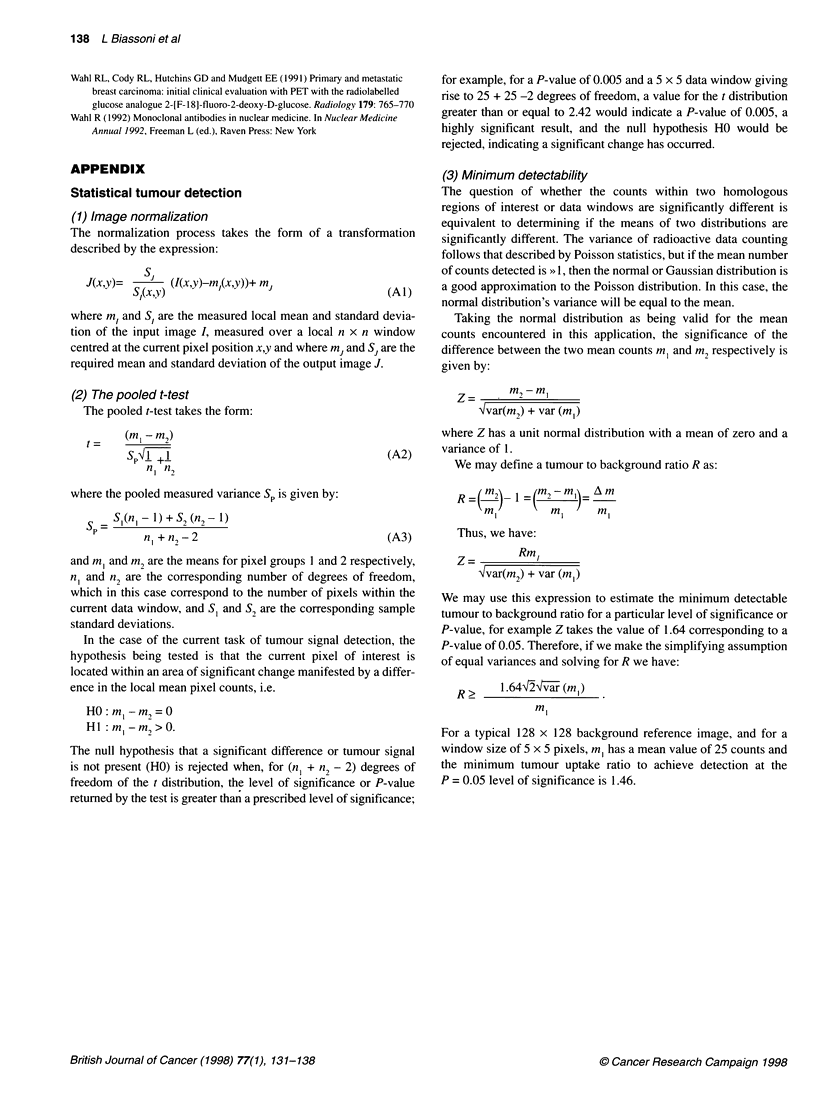# 99mTc-labelled SM3 in the preoperative evaluation of axillary lymph nodes and primary breast cancer with change detection statistical processing as an aid to tumour detection.

**DOI:** 10.1038/bjc.1998.21

**Published:** 1998

**Authors:** L. Biassoni, M. Granowska, M. J. Carroll, S. J. Mather, R. Howell, D. Ellison, F. A. MacNeill, C. A. Wells, R. Carpenter, K. E. Britton

**Affiliations:** Department of Nuclear Medicine and Research Laboratory, St Bartholomew's Hospital, The Royal Hospitals NHS Trust, London, UK.

## Abstract

**Images:**


*British Journal of Cancer* (1998) **77**(1), 131–138
© 1998 Cancer Research Campaign

### $^{99m}$Tc-labelled SM3 in the preoperative evaluation of axillary lymph nodes and primary breast cancer with change detection statistical processing as an aid to tumour detection

**L Biassoni[1], M Granowska[1], MJ Carroll[1], SJ Mather[1], R Howell[1], D Ellison[1], FA MacNeill[2], CA Wells[3], R Carpenter[2] and KE Britton[1]**

[1]Department of Nuclear Medicine and Research Laboratory, [2]Surgical Breast Unit, and [3]Department of Histopathology, St Bartholomew's Hospital, The Royal Hospitals NHS Trust, London, UK

**Summary** The extent of primary surgery for breast cancer could be tailored to the patient if previous information on the presence or absence of lymph node involvement could be reliably determined. Prospective radioimmunoscintigraphy in 29 patients with primary breast cancer that was found on screening has been undertaken with 555 MBq (15 mCi) $^{99m}$Tc SM3, an Imperial Cancer Research Fund (ICRF) murine monoclonal antibody, 0.5 mg with images at 10 min and 22 h, and analysis using a change detection algorithm. Sites of significant change between the early and later images were displayed as a map of probabilities. Image-positive and -negative axillary lymph nodes were compared by histology in the 28 evaluable patients. The correct identification of the presence or absence of node involvement, even if impalpable, has been shown in 24 out of 28 patients (29 lymph node groups). Sensitivity was 90% (nine out of ten), specificity 84% (16 out of 19) and accuracy 86%. These results encourage further assessment of this technique.

**Keywords:** radioimmunoscintigraphy: breast cancer; monoclonal antibody; change detection analysis; axillary lymph node

Surgeons have become more conservative in their approach towards patients with breast cancer. The introduction of segmental mastectomy as a valid surgical option, combined with the mammographic detection of early, small cancers of the breast, has led to questioning the need for axillary lymph node dissection in every patient.

Some surgeons feel that axillary lymph node dissection should be performed in all patients with breast cancer to stage the disease and to guarantee local control in the axillae and believe that it has a prognostic and therapeutic role (Harris and Osteen, 1985). Selection for chemotherapy is based on axillary node status. Surgery is currently the only reliable way of assessing nodal disease; unfortunately, to obtain this information, those women with node-negative disease receive unnecessarily extensive surgery. Another group of surgeons consider that axillary lymph node dissection has only a prognostic aim. Although it is important to take away the axillary lymph nodes that are macroscopically infiltrated by tumour, they consider that micrometastases do not produce recurrence in the axilla or affect overall survival (Fisher et al, 1985).

Axillary lymph node dissection has already been abandoned for ductal carcinoma in situ (DCIS) because of the extremely low rate of lymph node metastases (Deckers, 1991). Routine node dissection for lesions larger than DCIS but with extremely low likelihood of axillary node involvement could also be abandoned, particularly if a preoperative imaging technique were available to tailor the extent of axillary surgery to the individual woman.

*Received 27 February 1997*
*Revised 25 June 1997*
*Accepted 3 July 1997*

*Correspondence to:* KE Britton, Department of Nuclear Medicine, St Bartholomew's Hospital, The Royal Hospitals NHS Trust, West Smithfield, London EC1A 7BE, UK

Radioimmunoscintigraphy has been used with success in the management of some neoplasms in specific clinical conditions (Britton and Granowska, 1987; Bischof Delaloye et al, 1989; Doerr et al, 1990; Massuger et al, 1990; Wahl, 1992; Britton et al, 1993; Granowska et al, 1993), but it is limited by low tumour to background ratio, low percentage of injected dose per gram taken up by the tumour and heterogeneity of expression of the antigen. To overcome these problems, a change detection algorithm has been applied to detect small changes with time in antibody uptake. This exploits the kinetics of the antibody, whose specific uptake increases with time, whereas non-specific uptake decreases with time after an initial distribution.

The aim of this study is to evaluate technetium-99m-labelled SM3 (stripped mucin 3), a monoclonal antibody produced by the Imperial Cancer Research Fund, for the purpose of evaluating preoperatively axillary lymph node involvement in patients with breast cancer that was found by a screening programme.

### MATERIALS AND METHODS

### Patients

A total of 29 women with recently diagnosed breast cancer from the national screening programme were studied. Their age ranged between 34 and 84 years (median 58 years). All had previous clinical examination, mammography, and fine-needle aspiration cytology (FNAC); some also had previous breast ultrasound (US) and excisional biopsy. All were preoperative patients and had given signed informed consent, after the Royal Hospitals Trust Ethics committee requirements.

### Monoclonal antibody

SM3, an IgG1 subclass murine monoclonal antibody, is one of the new antibodies that are reactive with the protein core of the polymorphic epithelial mucin (PEM) series of antigens in the ducts of the breast. It binds specifically to the partially deglycosylated mucin that is typical of the cancer cells (Girling et al, 1989) and is usually unreactive with the fully processed mucin produced by the normal and lactating mammary gland (Burchell et al, 1987). An immunohistochemical study of breast tumours and tissues has shown that the SM3 antibody reacts strongly with the majority of primary breast cancers (91%) but shows little or no reaction with benign breast tumours, resting or lactating breast and most normal tissues (Burchell and Taylor-Papadimitriou, 1993). In normal breast epithelial cells, the SM3 epitope is masked by the carbohydrate side-chains.

### The radiolabel

Technetium-99m forms stable complexes with sulphur-containing compounds and can be made to bind to the sulphur-containing amino acids in the immunoglobulin. Normally, these thiol groups are in the oxidized, disulphide form and are unavailable for labelling. However, in the presence of mild reducing conditions, free thiols can be exposed to form a stable complex with technetium-99m (Mather and Ellison, 1990).

The antibody was concentrated by ultrafiltration to approximately 10 mg $ml^{-1}$. To a stirred solution of antibody, sufficient 2-mercaptoethanol was added to provide a molar ratio of 1000:1 2-mercaptoethanol–antibody. The mixture was incubated at room temperature for 30 min with continuous rotation, and the reduced antibody was purified by gel filtration on Sephadex–G5O using phosphate-buffered saline as a mobile phase. The antibody fractions were collated and divided into 0.5-mg aliquots. These were frozen immediately at –10°C and stored for further use.

When imaging was required, an antibody aliquot was thawed and an Amerscan methylene diphosphonate, MDP, kit was reconstituted with 5 ml of 0.9% sterile saline. An aliquot (50 μl) of MDP solution was added, followed by $^{99m}$Tc pertechnetate, 16 mCi (600 MBq) to the antibody/MDP mixture, which was gently shaken for 10 min. The labelling efficiency was assessed by thin-layer chromatography (ITLC) developed in 0.9% saline and confirmed to be over 95%. The labelled antibody is stable up to 24 hours in vitro after preparation. The antigen-binding ability of the radiolabelled antibody was compared to the unlabelled starting material (relative immunoreactivity) by testing with an enzyme-linked immunosorbent assay (ELISA). The average value was 75%. The technetium-99m-labelled antibody is stable in vivo. There was no thyroid uptake of $^{99m}$Tc even after 24 h in patients who had received no thyroid blocking medication.

### Injection and acquisition

The radiopharmaceutical [15 mCi (555 MBq), 0.5 mg in 1 ml] was injected in the antecubital vein of the arm opposite the lesion. Early images were acquired 10 min after the injection and included both breasts, both axillae, the heart and major vessels and part of the liver. After making small indelible ink marks on the skin, tiny $^{57}$Co sources (Amersham International) were positioned on the sternal notch, xiphisternum, axillary tail of both breasts and lower costal margins on the hemiclavicular line. The gamma-camera (Siemens Orbiter 75) with a low-energy, parallel-hole general-purpose collimator was linked to a Micas V computer (Park Medical). The camera was peaked on 122 keV with a 15% window and an image was acquired for 60 s. The position of each marker was recorded on a blank film superimposed on the screen of the computer before the beginning of the acquisition; this helped in the repositioning of the camera the following day for the 24 h images. Another picture was acquired for 60 s with the patient wearing a flexible lead strip giving the inferior outlines of the breasts and with cobalt markers corresponding to both nipples. The main image of the patient lying in the same position, with the markers and the breast outline off, was acquired with the camera set on the technetium-99m 140-keV peak with a 15% window. Acquisition stopped at 800 Kcounts (2000 Kcounts in the last eight patients). This image was used as a template in the processing with the change detection algorithm, demonstrating initial vascular and non-specific activity. The other two images provided anatomical references to aid in the registration of the 10-min and 22-h images. A right lateral and a left lateral picture of the upper chest and axilla were also acquired for 500 Kcounts. These images were taken to have a further confirmation of the presence of any lesion, and were preceded by a picture with $^{57}$Co skin markers positioned on the axillary tail, xiphisternum, inferior costal margin and posterior aspect of the axilla with the camera settings and acquisition as before. For all images the matrix size was 256 × 256.

At 22 h, the same set of pictures was acquired, the anterior image taking about 20 min, with the patient in the same position. The blank film used on the previous day to record the position of the skin markers was positioned on the screen of the computer again and the camera was positioned in such a way that the skin markers of the 22-h image matched their position as recorded on the blank film of the 10-min image; thus, satisfactory initial patient registration could be obtained and subsequently improved by computer analysis. This is required for the correct application of the change detection algorithm.

### Significance probability mapping

The significance probability mapping (SPM) for this study was based on a statistical pixel by pixel comparison between the 10-min and the 22-h images. Before statistical pixel comparison an image normalization or prewhitening stage is carried out. The image normalization process ensures a significant pixel to pixel decorrelating effect and therefore the independence requirement of a chosen statistical test is largely met (see Appendix, part 1, for the formula). The derived map is called statistical scaling and is related to the image processing techniques of unsharp masking. A window, five by five pixels in extent, has been chosen for the current application. For the two homologous images, a small centralized window size is typically five by five pixels square. For each of these small subpopulations a parametric statistical test, the pooled *t*-test, is used to test the significance of any difference in the mean counts of the windowed pixel subset (see also Appendix, parts 2 and 3). The SPM parametric image is generated by this process.

The various *P*-values are presented as a colour table, so that the resulting image is a colour map of *P*-values each indicating the significance of the change that has occurred between the 10-min and 22-h image.

### Interpretation of scans

Planar and processed images were interpreted by two nuclear medicine physicians who were unaware of the clinical, surgical

© Cancer Research Campaign 1998

**Table 1** Comparison of $^{99m}$Tc SM3 immune scan results with clinical and histopathological data

| Patient number | Name | Clinical | | Planar | | Probability map | | Pathology | | | | Result | |
|---|---|---|---|---|---|---|---|---|---|---|---|---|---|
| | | B | N | B | N | B | N | B | T size (mm) | *n* | Histology | B | N |
| 1 | HN | R | – | R | + | R;B4 | +;N5 | R;G1 | 21 | 1/16 | Duct | TP | TP |
| 2 | MOM | L | – | L | – | L;B5 | +;N4 | L;G2 | 21 | 5/15 | Duct | TP | TP |
| 3 | LS | L | + | L | – | L;B5 | +;N3 | L;G1 | 22 | 0/7 | Duct + lobul | TP | FP |
| 4 | MC | R | – | – | – | – | – | R | | 0/9 | Muc | FN | TN |
| 5 | MD | Rec | – | R | – | R;B5 | – | R;G3 | | 0/1 | Duct | TP | TN |
| 6 | MG | bxR | – | | | | | bx:G1 | | | DCIS | | |
| | | | | – | – | R;B2 | – | no res | | 0/10 | – | TN | TN |
| 7 | DG | R | – | – | – | R;B5 | +;N5 | R;G3 | 12 | 1/7 | Duct | TP | TP |
| | | | | – | – | R;B5 | | R;G3 | 20 | | Duct | TP | |
| 8 | TC | N | – | R | – | R;B4 | – | R | | 0/9 | DCIS | TP | TN |
| 9 | CS | R | – | R | – | R;B5 | – | R;G2 | 18 | 0/7 | Duct + lobul | TP | TN |
| 10 | JW | L | – | L | + | L;B5 | +;N5 | L;G3 | 23 | 3/19 | Duct | TP | FP |
| 11 | MM | R | + | R | – | R;B4 | +;N5 | bx * | | ND | Lobul + duct | TP | ND |
| 12 | AS | R | – | – | + | R;B3 | +;N5 | R;G2 | 17 | 5/21 | Duct | FN | TP |
| | | L | – | L | + | L;B4 | +;N5 | bx- | | ND | Fibr | FP | ND |
| 13 | RH | bx | – | | | | | bx:G3 | 22 | – | Duct | | |
| | | R | | R | – | R;B5 | – | no res | | 0/18 | | FP | TN |
| 14 | SF | bx | – | R | | R;B5 | – | papill | | | | FP | |
| | | L | | L | – | L;B5 | – | bx:G2 | | | | | |
| | | | | | | | | L:+ | 22 | 1/14 | Duct + lobul | TP | FN |
| 15 | CH | R | – | R | – | R;B5 | N3 | R;G3 | 13 | 0/22 | Duct | TP | FP |
| 16 | TM | bxR | – | | | | | bx:G2 | 15 | | Duct | | |
| | | | | – | – | R;B4 | – | R:+ | 1.4 | 0/25 | | FP | TN |
| 17 | GS | R | – | R | – | R;B5 | – | R;G1 | 35 | 0/2 | DCIS | TP | TN |
| 18 | EB | R | – | – | – | R;B5 | +;N5 | R;G1 | 21 | 1/16 | Duct | TP | TP |
| 19 | CMK | L | + | L | – | L;B4 | +;N5 | R;G2 | 25 | 6/13 | Duct | TP | TP |
| 20 | WH | L | – | – | – | L;B5 | +;N5 | L;G1 | 15 | 0/9 | Duct | TP | FP |
| 21 | NS | L | – | L | – | L;B5 | – | L;G2 | 3 | 0/14 | Duct | TP | TN |
| | | | | | | | | | + | | + | | |
| | | | | | | | | | 20 | | DCIS | | |
| 22 | AB | bx | – | | | | | bx:G1 | | | DCIS | | |
| | | R | | R | – | R;B4 | – | no res | | 0/9 | | FP | TN |
| 23 | JG | L | + | L | – | L;B5 | – | L;G3 | 21 | 0/14 | duct | TP | TN |
| | | R | – | – | – | R;B5 | – | R;G3 | 15 | 0/12 | lobul | TP | TN |
| 24 | KS | R | – | R | – | R;B5 | – | R;G2 | 13.5 | 0/14 | duct | TP | TN |
| | | | | | | | | | | | + | | |
| | | | | | | | | | | | DCIS | | |
| 25 | DW | R | – | R | – | R;B5 | +;N4 | R;G2 | 20 | 5/14 | duct | TP | TP |
| | | | | | | | | | | | + | | |
| | | | | | | | | | | | DCIS | | |
| | | | | | | R;B5 | | | | | papill | FP | |
| 26 | AN | L | + | L | – | L;B5 | +;N4 | L;G2 | 38 | 4/18 | duct | TP | TP |
| | | | | | | | | | | | + | | |
| | | | | | | | | | | | DCIS | | |
| 27 | MM | R | – | R | – | R;B5 | – | R;G3 | 13 | 0/9 | duct | TP | TN |
| | | | | | | | | | | | + | | |
| | | | | | | | | | | | DCIS | | |
| 28 | AB | L | – | – | – | L;B1 | – | L;G2 | 12 | 0/11 | duct | FN | TN |
| | | | | | | | | | | | + | | |
| | | | | | | | | | | | DCIS | | |
| 29 | MOC | R | – | – | – | R;B1 | – | R;G1 | 12 | 0/9 | duct | FN | TN |
| | | | | | | | | | | | + | | |
| | | | | | | | | | | | DCIS | | |

Abbreviations: B, breast; N, nodes; Mx, mammography; PROB MAP, probability mapping; Histol, histology; R, right; L, left; G1, 2, 3, grading of differentiation (G1, well-differentiated; G2, moderately differentiated; G3, poorly differentiated); B1–B5, grading of breast immune scan report; N1–5, grading of node immune report; TP, true positive; TN, true negative; FP, false negative; FN, false negative; bx, biopsy, DCIS, ductal carcinoma in situ; duct, ductal; lobul, lobular; muc, mucinous; rec, recurrence; ND, not done; no res, no residual; papill, papilloma; * pt operated elsewhere, histology not available; fibr, fibrocystic changes with calcification. Grading of reports: B1, N1, R1, normal; B2, N2, R2, equivocal–normal; B3, N3, R3, equivocal; B4, N4, R4, abnormal; B5, N5, R5, definitely abnormal.

and histological data. Results of the probability mapping were graded from 1 to 5, according to the colour map blue green/orange red/bright red related to the number of standard deviations of difference between the two images: 1 (corresponding to the blue) was considered normal; 2 meant that the result was probably normal (the difference in the number of counts between the image at 10 min and the image at 22 h was up to one standard deviation); 3 meant 1–2 standard deviations; 4 probably abnormal (difference was 2–3 standard deviations); 5 (corresponding to the bright red) was definitely abnormal. The difference in the number of counts

**Figure 1** Patient number 10 (JW). $^{99m}$Tc SM3 radioimmunoscintigraphy. Anterior views of the chest at 10 min (top left) and 24 h (top right). Change detection probability map showing significant differences (bottom left) and key to outlines (bottom right). Planar images show cardiac blood pool and liver, and a small blue focus can be seen in the region of the left breast at 24 h and possibly in the left axilla. The change detection map shows the significance of the differences between the 24-h image and the 10-min image. $P < 0.001$ in red, $P < 0.01$ in orange, $P < 0.05$ in green and $P > 0.05$ in blue. A highly significant focal area of increased uptake is seen in the left breast corresponding to the tumour, in the left axilla corresponding to involved nodes and in the internal mammary region on the left (not sampled at surgery). Breast surgery confirmed a primary left ductal carcinoma and that 3 out of 19 nodes sampled from the left axilla were involved between the image at 10 min and image at 22 h was more than 3 standard deviations. Findings from the probability mapping was also compared with other areas of non-significant uptake, owing to activity in blood vessels or to imperfect repositioning of the patient during the 22-h image.

Areas above the brachial and subclavian arteries, even if the change detection algorithm discovered significant increase with time of radiolabelled antibody, were not considered because of the inability of the patient to control small head and neck movements that cause artefacts in this region.

An increase in activity with time in the liver was usual, owing to the uptake and metabolism of the antibody, so the organ was isolated in a region of interest and ignored. Areas of negative change such as the heart and big vessels were also excluded. These were isolated in regions of interest and not considered.

### RESULTS

The clinical and imaging, surgical and histopathological data are set out in Table 1. The investigated patients had 346 surgically removed lymph nodes from 29 axillae (one patient, no. 11, was operated in another hospital and histology was not made available and the date excluded; another patient, no. 23, had bilateral mastectomy with bilateral axillary clearance).

### Toxicity

The radioimmunoscintigraphy was extremely well tolerated, with no discomfort. No patient complained of later arthralgia, or rash, and there was no serum sickness.

### Imaging results

Immunoscintigraphic findings (planar images with and without change detection algorithm with probability mapping) were compared with the clinical examination and with histopathology (29 axillae in 28 evaluable patients) (Figures 1 and 2).

Planar images of 29 axillary lymph node regions studied showed 3 out of ten true positives and 18 out of 19 true negatives, with seven out of ten false negatives and 1 false positive out of 19. Sensitivity was 30%, specificity 95% and accuracy 72%.

Change detection algorithm with probability mapping of the 29 axillary lymph node groups showed nine out of ten true positives, 16 out of 19 true negatives, 3 out of 19 false positives and one false negative out of ten. Sensitivity was 90%, specificity 84% and accuracy 86%. The three false positives in the axilla were due to a node that had been called positive when it was due to tumour extension (patient no. 15); movement artefact interpreted as node positive (patient no. 20); and finding a node positive deep in axilla, which may have been taken for tumoral venous invasion (demonstrated

© Cancer Research Campaign 1998

**Figure 2** Patient number 24 (KS). $^{99m}$Tc SM3 radioimmunoscintigraphy anterior chest views. Key as for Figure 1. Planar images show at 24 h some possible blue activity in the region of the right breast. Change detection map shows highly significant uptake in the right breast, non-significant uptake in the left breast and no uptake in either axilla. A primary ductal carcinoma was confirmed at surgery and none of 14 nodes was shown to be involved subsequently at the histopathological examination – patient no. 3) or may not have been included in the axillary clearance. The one false negative was due to a micrometastasis in 1 out of 20 nodes sampled (patient no. 14).

Planar images of 35 breast lesions in 29 patients (29 preoperative breast cancers, two benign papillomas, three breasts studied after the excisional biopsy, one breast with fibrocystic changes and involution with calcification) showed 18 out of 29 true positives (62% sensitivity), no true negatives, 10 out of 29 false negatives and six false positives.

The change detection algorithm with probability mapping of the breast showed 25 out of 29 true positives (sensitivity 86%), no true negative, six false positives and four false negatives. The six false positives in the breast were due to: excisional biopsy performed 30–50 days before the scan (three patients: no. 6, no. 13 and no. 22); papilloma with areas of apocrine metaplasia, which showed immunocytochemical uptake of SM3 in two patients (no. 14 and no. 25); and fibrocystic changes and involution with calcification, demonstrated after biopsing a breast area that was suspicious at mammography, which showed uptake of SM3 (one patient, no. 12). The four false negatives in the breast were due to: mucinous carcinoma (the abundant secretion of mucin may have prevented the antibody from getting to the tumour (patient no. 4); an equivocal uptake (grade 3) was reported as negative in one patient (no. 12); and primary tumours (ductal carcinomas + ductal carcinoma in situ) of 12 mm were missed both by the planar images and the change detection algorithm (patients nos 28 and 29).

### DISCUSSION

This pilot study had the specific aim of evaluating metastatic involvement of axillary lymph nodes in patients with breast cancer and, as a secondary aim, evaluating the uptake of the radiolabelled antibody in the primary tumour. For this reason, the supine position was chosen to be more appropriate for the study of the lymph nodes rather than for breast imaging.

The key principles of the study can be summarized as follows:

- Specific uptake of the antibody increases with time, whereas non-specific uptake decreases with time; this explains why the 10 min image, which reflects vascular activity and non-specific uptake with no significant specific monoclonal antibody uptake, is important as a template with which to compare the later images.
- Imaging with $^{99m}$Tc gives a powerful signal of antibody uptake in spite of the background activity. The more potent the signal, the higher the signal-to-noise ratio and the less the noise is inherent in the signal (Britton and Granowska, 1987). SM3 is a good antibody for studying breast cancer (Granowska et al, 1996) as well as ovarian cancer (Granowska et al, 1993).

Previous studies in ovarian cancer using $^{99m}$Tc SM3 have shown less than 20% human anti-mouse antibodies and no clinical reactions (Granowska et al, 1990). They were not evaluated here.

SPM is the process of deriving a parametric image whose elements contain the significance $P$-value that is returned after

© Cancer Research Campaign 1998

performing a parametric test between the pixels of the two images being compared. There are two distinct approaches to SPM, i.e. global linear regression and pixel by pixel statistical comparison. The *global linear regression approach* uses a two-dimensional co-occurrence matrix or scattergraph and has been used successfully in ovarian cancer (Granowska et al, 1988).

Because of its limitations, a new approach was developed using a SPM based on a *statistical pixel by pixel comparison* between the 10 min and the 22-h images. The image normalization (prewhitening) performed before the statistical pixel comparison, besides aiding in the detection process directly, overcomes the problem of interpixel correlation.

The change detection algorithm is essential for detecting changes in uptake with time. A typical biological half-life of clearance of $^{99m}$Tc-labelled SM3 whole antibody from the blood is 24 h. For a whole antibody and a typical compact, reasonably vascular, tumour, about 75% of the total uptake would be expected to occur in the first 12 h. Images taken at 10 min and 22–24 h cover, therefore, a long enough period for a significant increase in uptake to occur over time.

In evaluating the results of the images of the uptake in the lymph nodes produced by the change detection algorithm, attention was paid to the pretest likelihood of axillary node involvement, that is considering the size of the primary tumour, whether the nodes were palpable on the clinical examination (Table 1).

The results obtained in axillary nodes comparing radioimmunoscintigraphy with surgical findings and histology in terms of sensitivity (90%) and specificity (84%) are encouraging. In this series of patients, clinical evaluation of nodal status was poor. Of five patients with palpable nodes, histology was positive in two, negative in two and not done in one. A total of seven out of nine patients with clinically impalpable nodes had a tumour present (Table 1).

Misregistration led to one false-positive result. The registration procedure has been improved. Provided the protocol is followed, the change detection algorithm is not particularly sensitive to movement. On the 10-min image, if a large background region is used for each $5 \times 5$ pixel region and compared with a small tumour within a region of $5 \times 5$ pixels at 22 h this will not be affected by a misregistration of one or two pixels. Any major misregistration is detected by 'negative shadows': negative deviations seen on the edges of active areas such as blood vessels, the heart and the liver.

The minimum detectable signal was shown to be an absolute tumour count of 37 c.p.s. per pixel over a typical background count of 25 c.p.s. per pixel with the minimal detectable tumour to background ratio of 1.46, which gives a difference significant at $P < 0.05$. The basis of the calculation is shown in the Appendix.

Three patients (nos 6, 13 and 22), who had excisional biopsy of the breast performed 30–55 days before the scan and who, at a subsequent operation, were demonstrated to be free of residual malignancy showed significant uptake of antibody in the area of the excisional biopsy. The reason for this is not clear: one hypothesis is that the uptake may be due to the new-grown tissue in the area of the surgical scar, whose young cell-surface glycoproteins may not have been entirely glycosylated and may therefore still expose the antigenic epitope. Two patients (nos 14 and 25) with images that were false positive for primary breast cancer had some areas of papillomatosis with apocrine metaplasia, which took up the antibody, as described by Burchell et al (1987).

The study appears to be in line with the recent literature on the topic. Tjandra et al (1989) reported immunolymphoscintigraphic findings in 40 patients with histologically proven or cytologically suspicious breast cancer. The authors conclude that, as involvement of axillary lymph nodes is of importance as a prognostic factor and as an indicator for adjuvant therapy, further improvement in diagnostic accuracy is needed before axillary dissection and histology can be replaced by immunolymphoscintigraphy scanning of the axillae. They can not explain the mechanism of non-specific uptake of antibody by normal lymph nodes or the mechanisms of action of a second blocking antibody.

Schatten et al (1994) made use of two $^{123}$I-radiolabelled monoclonal antibodies, one specific and the other non-specific for breast cancer, with a 1-week interval between injections. Comparisons of both pictures, with possible specific and non-specific uptake, was obtained. A typical pitfall of subcutaneous immunoscintigraphy is described as the inaccessibility of the lymph nodes attributable to the occlusion of the lymphatic vessels, which is frequently the case when a metastasis breaks through the lymph node capsule.

Previous studies in breast cancer (Deland et al, 1979) with anti-CEA antibodies are not ideal because CEA antigen is released by the cancer cell and can be trapped in the draining lymph nodes, which bind the antibody so that the node may appear positive in imaging even when no cancer cells are present, as noted in colorectal cancer (Granowska et al, 1989).

Preliminary results from McEwan et al (1994) in 53 patients with breast cancer, with a $^{99m}$Tc-labelled monoclonal antibody (170 H.82), planar and SPET imaging show a sensitivity of 90% and a positive predictive value of 95% for locoregional disease (breast and axillary lymph nodes, many of which were palpable). This differs from the patient studies reported here, which were from a national screening programme, mostly with impalpable axillary nodes (Table 1).

Apart from monoclonal antibodies, other radiopharmaceuticals are being studied. $^{99m}$Tc-MIBI (methoxy-isobutyl-isonitrile) is being used in the evaluation of breast cancer and axillary lymph nodes with good results (Khalkhali et al, 1994; Palmedo et al, 1996). However, muscle uptake of MIBI may interfere with the evaluation of the axilla. Positron emission tomographic (PET) scans showing increased $^{18}$FDG (fluorodeoxyglucose) uptake in axillary nodes have a strong likelihood of detecting cancer, although FDG uptake may also occur in reactive hyperplasia. Avril et al (1995) evaluated the diagnostic accuracy of axillary PET imaging in patients with recently detected breast tumours. $^{18}$FDG PET showed a sensitivity of 72% and a specificity of 96%. Five false-negative results were classified as pN1 at histology and lymph node size was smaller than 2 cm. The conclusion was that axillary node dissection was still required, even in patients with negative axillary PET scans, and further improvements in spatial resolution were required to increase sensitivity of PET imaging. In the study by Wahl et al (1991), ten out of ten primary tumours were visualized and FDG scanning was reliable in the detection of lymph node and distant metastases. Nieweg et al (1993), found that monitoring treatment with PET FDG in 10 out of 11 patients with primary breast cancer the tumour was visualized. In all five patients with increased uptake in the lymph nodes, pathological proof of metastatic cancer was found. Adler et al (1993) correctly found ten out of ten axillary nodes to be negative and nine out of ten to be positive using FDG PET; however, in the positive axillae five out of those nine had 10–26 nodes involved that were probably clinically evident, although this was not stated.

These preliminary results with radioimmunoscintigraphy allow the following conclusions:

© Cancer Research Campaign 1998

a. planar images are insufficiently sensitive for the detection of involved impalpable or small axillary lymph nodes; and
b. $^{99m}$Tc-labelled SM3 in conjunction with probability mapping appears a promising technique for the imaging of the axilla: clinically negative lymph nodes can be shown correctly positive; and clinically positive nodes can be shown correctly negative. A good specificity is particularly important because false negatives are not well considered, whereas false positives are much better tolerated.

Previous knowledge about the involvement of axillary lymph nodes should enable the extent of surgery to be tailored to the individual woman. A more generous use of axillary surgery could be advised in node-positive patients and a full axillary clearance may be avoided in node-negative patients, in whom sentinel node sampling at surgery might be sufficient (Krag et al, 1993). The patient could then be spared the complications deriving from axillary node dissection. This study with $^{99m}$Tc SM3 and the use of a change detection algorithm is encouraging in the pursuit of this goal.

The results obtained suggest the prospective evaluation of a surgical strategy of axillary clearance for patients with positive lymph nodes at the $^{99m}$Tc SM3 immunoscintigraphy with change detection statistical processing, and axillary sampling in patients with negative lymph nodes.

### ACKNOWLEDGEMENTS

We are grateful to Miss Nish Fernando and Mrs Cherry Sebastian for the acquisition of the scans, and to Mrs Margaret Sullivan for her help in the recruitment of the patients. This work has been supported by a grant from Cytogen Corporation, USA. We also acknowledge the support of the Imperial Cancer Research Fund and are grateful for the use of the facilities of the St Bartholomew's Hospital Foundation for Research. We thank especially Dr Joy Burchell and Dr Joyce Taylor-Papadimitriou for making available to us their knowledge and expertise on SM3.

### REFERENCES

Adler LP, Crowe JP, Al-Kaisai NK and Sunshine JL (1993) Evaluation of breast masses and axillary lymph nodes with [F-18] 2-deoxy-2-fluoro-D-glucose PET. *Radiology* **187**: 743–750

Avril N, Janicke F, Dose J, Ziegler S, Bense S, Zincke M, Romer W, Weber W, Herz M and Schwaiger M (1995) Evaluation of axillary lymph node involvement in breast cancer patients using F-18 FDG PET. *Eur J Nucl Med* **22**: 733

Biersack HJ (1995) Mammoscintigraphy with Tc-99m MIBI in patients with suspicious breast nodules: a comparison of planar and SPECT imaging techniques. *Eur J Nucl Med* **22**: 725

Bischof-Delaloye A, Delaloye B, Buchegger F, Gilgien W, Studer A, Curchod S, Givel JC, Mosimann F, Pettavil J and Mach J-P (1989) Clinical value of immunoscintigraphy in colorectal carcinoma patients: a prospective trial. *J Nucl Med* **30**: 1646–1656

Britton KE and Granowska M (1987) Radioimmunoscintigraphy in tumour identification. *Cancer Surv* **6**: 1247–1267

Britton KE and Granowska M (1993) The present and the future of radiolabelled antibodies in oncology. *Ann Nucl Med* **7**: 127–132

Burchell J, Gendler S, Taylor-Papadimitriou J, Girling A, Lewis A, Millis R and Lamport D (1987) Development and characterization of breast cancer reactive monoclonal antibodies directed to the core protein of the human milk mucin. *Cancer Res* **47**: 5476–5482

Burchell J and Taylor-Papadimitriou J (1993) Effect of modification of carbohydrate side chains on the reactivity of antibodies with core-protein epitopes of the MUC1 gene product. *Epith Cell Biol* **2**: 155–162

Deckers PJ (1991) Axillary dissection in breast cancer: when, why, how much, and for how long? Are these operations soon to be extinct? *J Surg Oncol* **48**: 217–219

Deland FH, Kim BE, Corgan RL, Casper FS, Primus FJ, Spremulli E, Estes N and Goldenberg DM (1979) Axillary lymphoscintigraphy by radioimmunodetection of carcino-embryonic antigen in breast cancer. *J Nucl Med* **20**: 1243–1250

Doerr RJ, Abdel-Nabi H, Baker JM and Steinberg S (1990) Detection of primary colorectal cancer using indium-111 monoclonal antibody B72.3. *Arch Surg* **125**: 1601–1605

Fisher B, Redmond C, Fisher ER, Bauer M, Wolmark N, Wickerham L, Deutsch M, Montegue E, Margolese R and Foster R (1985) Ten year results of a randomized clinical trial comparing radical mastectomy and total mastectomy with or without radiation. *N Engl J Med* **312**: 674–681

Girling A, Bartkova J, Burchell J, Gendler S, Gillet C and Taylor-Papadimitriou J (1989) A core protein epitope of the polymorphic epithelial mucin detected by the monoclonal antibody SM3 is selectively exposed in a range of primary carcinomas. *Int J Cancer* **43**: 1072–1076

Goldenberg DM and Larson SM (1992) Radioimmunodetection in cancer identification. *J Nucl Med* **33**: 803–814

Granowska M, Nimmon CC, Britton KE, Crowther M, Mather SJ, Slevin ML and Shepherd JH (1988) Kinetic analysis and probability mapping applied to the detection of ovarian cancer by radioimmunoscintigraphy. *J Nucl Med* **29**: 599–607

Granowska M, Jass JR, Britton KE and Northover JMA (1989) A prospective study of the use of 111In-labelled monoclonal antibody against carcinoembryonic antigen in colorectal cancer and of some biological factors affecting its uptake. *Int J Colorect Dis* **4**: 97–108

Granowska M, Mather SJ, Jobling T, Naeem M, Birchall J, Taylor Papadimitriou J, Shepherd J and Britton KE (1990) Radiolabelled stripped mucin, SM3, monoclonal antibody for immunoscintigraphy of ovarian tumours. *Int J Biol Mark* **5**: 89–96

Granowska M, Britton KE, Mather SJ, Lowe DG, Ellison D, Bomanji J, Birchall J, Taylor Papadimitriou J, Hudson CR and Shepherd JH (1993) Radioimmunoscintigraphy with technetium-99m labelled monoclonal antibody, SM3, in gynaecological cancer. *Eur J Nucl Med* **20**: 483–488

Granowska M, Biasoni L, Carroll MJ, Howell R, Mather SJ, Ellison D, Granowski A and Britton KE (1996) Breast cancer $^{99m}$Tc SM3 Radioimmunoscintigraphy. *Acta Oncol* **35**: 319–321

Harris JR and Osteen RT (1985) Patients with early breast cancer benefit from effective axillary treatment. *Breast Cancer Res Treat* **5**: 17–21

Khalkhali I, Mena I, Jouanne E, Diggles L, Venegas R, Block J, Alle K and Klein S (1994) Prone scintimammography in patients with suspicion of breast cancer. *J Am Coll Surg* **178**: 491–497

Krag DN, Weaver DL, Alex JC and Fairbank JT (1993) Surgical resection and radiolocalization of the sentinel lymph node in breast cancer using a gamma probe. *Surg Oncol* **2**: 335–340

McEwan AJB, Akram I, Boniface G, Golberg L, McQuarrie SA, Golberg K, Amyotte G, Hornig B, Noujaim AA and MacLean GD (1994) Tc-99m monoclonal antibody in the evaluation of locoregional disease in patients with breast cancer. *Eur J Nucl Med* **21** (suppl.): S15

Massuger LFAG, Keenemans P, Claessens RAMJ et al (1990) Immunoscintigraphy of ovarian cancer using indium-111 labelled OV-TL3 F(ab′)2 monoclonal antibody. *J Nucl Med* **31**: 1802–1810

Mather SJ and Ellison D (1990) Reduction-mediated Tc-99m labelling of monoclonal antibodies. *J Nucl Med* **31**: 692–697

Nieweg OE, Kim EE, Wong WH, Broussard WF, Singletary SE and Tilbury RS (1993) Positron Emission Tomography with fluorine-18-deoxyglucose in the detection and staging of breast cancer. *Cancer* **71**: 3920–3925

Palmedo H, Grunwald F, Bender H, Schomburg A, Mallmann P, Krebs B and Biersack HJ (1996) Scintimammography with Technetium-99m methoxyisobutylisonitrile: comparison with mammography and magnetic resonance imaging. *Eur J Nucl Med* **23**: 940–946

Silverstein MJ, Giersone D, Waisman JR, Senofsky GM, Colburn WJ and Gamagami P (1994) Axillary lymph node dissection for T1a breast carcinoma – Is it indicated? *Cancer* **73**: 664–667

Schatten C, Bawada M, Mandeville R, Enzelsberger H, Angelberger P, Czerwenka K, Kubista E and Pateisky N (1994) Combined use of 123-I labelled BCDF9 and 4C4 monoclonal antibodies with dissimilar specificity for breast cancer: implication for detection limits of immunolymphoscintigraphy in the assessment of axillary lymph nodes. *Nucl Med Comm* **15**: 422–429

Tjandra JJ, Russell IS, Collins JP, Andrews JT, Lichtenstein M, Binns D and McKenzie IFC (1989) Immunolymphoscintigraphy for the detection of lymph node metastases from breast carcinoma. *Cancer Res* **49**: 1600–1608

© Cancer Research Campaign 1998

Wahl RL, Cody RL, Hutchins GD and Mudgett EE (1991) Primary and metastatic breast carcinoma: initial clinical evaluation with PET with the radiolabelled glucose analogue 2-[F-18]-fluoro-2-deoxy-D-glucose. *Radiology* **179**: 765–770

Wahl R (1992) Monoclonal antibodies in nuclear medicine. In *Nuclear Medicine Annual 1992*, Freeman L (ed.), Raven Press: New York

### APPENDIX

### Statistical tumour detection

#### *(1) Image normalization*

The normalization process takes the form of a transformation described by the expression:

$$J(x,y)= \frac{S_J}{S_I(x,y)} (I(x,y)-m_I(x,y))+ m_J \tag{A1}$$

where $m_I$ and $S_I$ are the measured local mean and standard deviation of the input image *I*, measured over a local $n \times n$ window centred at the current pixel position *x,y* and where $m_J$ and $S_J$ are the required mean and standard deviation of the output image *J*.

#### *(2) The pooled t-test*

The pooled *t*-test takes the form:

$$t = \frac{(m_1 - m_2)}{S_P\sqrt{\frac{1}{n_1}+\frac{1}{n_2}}} \tag{A2}$$

where the pooled measured variance $S_P$ is given by:

$$S_P = \frac{S_1(n_1 - 1) + S_2 (n_2 - 1)}{n_1 + n_2 - 2} \tag{A3}$$

and $m_1$ and $m_2$ are the means for pixel groups 1 and 2 respectively, $n_1$ and $n_2$ are the corresponding number of degrees of freedom, which in this case correspond to the number of pixels within the current data window, and $S_1$ and $S_2$ are the corresponding sample standard deviations.

In the case of the current task of tumour signal detection, the hypothesis being tested is that the current pixel of interest is located within an area of significant change manifested by a difference in the local mean pixel counts, i.e.

$$H0 : m_1 - m_2 = 0$$
$$H1 : m_1 - m_2 > 0.$$

The null hypothesis that a significant difference or tumour signal is not present (H0) is rejected when, for $(n_1 + n_2 - 2)$ degrees of freedom of the *t* distribution, the level of significance or *P*-value returned by the test is greater than a prescribed level of significance; for example, for a *P*-value of 0.005 and a $5 \times 5$ data window giving rise to 25 + 25 –2 degrees of freedom, a value for the *t* distribution greater than or equal to 2.42 would indicate a *P*-value of 0.005, a highly significant result, and the null hypothesis H0 would be rejected, indicating a significant change has occurred.

#### *(3) Minimum detectability*

The question of whether the counts within two homologous regions of interest or data windows are significantly different is equivalent to determining if the means of two distributions are significantly different. The variance of radioactive data counting follows that described by Poisson statistics, but if the mean number of counts detected is »1, then the normal or Gaussian distribution is a good approximation to the Poisson distribution. In this case, the normal distribution's variance will be equal to the mean.

Taking the normal distribution as being valid for the mean counts encountered in this application, the significance of the difference between the two mean counts $m_1$ and $m_2$ respectively is given by:

$$Z = \frac{m_2 - m_1}{\sqrt{\mathrm{var}(m_2) + \mathrm{var}\,(m_1)}}$$

where *Z* has a unit normal distribution with a mean of zero and a variance of 1.

We may define a tumour to background ratio *R* as:

$$R = \left(\frac{m_2}{m_1}\right) - 1 = \left(\frac{m_2 - m_1}{m_1}\right) = \frac{\Delta m}{m_1}$$

Thus, we have:

$$Z = \frac{Rm_1}{\sqrt{\mathrm{var}(m_2) + \mathrm{var}\,(m_1)}}$$

We may use this expression to estimate the minimum detectable tumour to background ratio for a particular level of significance or *P*-value, for example *Z* takes the value of 1.64 corresponding to a *P*-value of 0.05. Therefore, if we make the simplifying assumption of equal variances and solving for *R* we have:

$$R \geq \frac{1.64\sqrt{2}\sqrt{\mathrm{var}}\,(m_1)}{m_1}.$$

For a typical $128 \times 128$ background reference image, and for a window size of $5 \times 5$ pixels, $m_1$ has a mean value of 25 counts and the minimum tumour uptake ratio to achieve detection at the $P = 0.05$ level of significance is 1.46.

*© Cancer Research Campaign 1998*